# Landscape Factors Facilitating the Invasive Dynamics and Distribution of the Brown Marmorated Stink Bug, *Halyomorpha halys* (Hemiptera: Pentatomidae), after Arrival in the United States

**DOI:** 10.1371/journal.pone.0095691

**Published:** 2014-05-01

**Authors:** Adam M. Wallner, George C. Hamilton, Anne L. Nielsen, Noel Hahn, Edwin J. Green, Cesar R. Rodriguez-Saona

**Affiliations:** 1 United States Department of Agriculture – Animal and Plant Health Services – Plant Protection Quarantine, Plant Inspection Station, Miami, Florida, United States of America; 2 Department of Entomology, Rutgers University, New Brunswick, New Jersey, United States of America; 3 Department of Ecology, Evolution and Natural Resources, Rutgers University, New Brunswick, New Jersey, United States of America; United States Department of Agriculture, Beltsville Agricultural Research Center, United States of America

## Abstract

The brown marmorated stink bug, *Halyomorpha halys*, a native of Asia, has become a serious invasive pest in the USA. *H. halys* was first detected in the USA in the mid 1990s, dispersing to over 41 other states. Since 1998, *H. halys* has spread throughout New Jersey, becoming an important pest of agriculture, and a major nuisance in urban developments. In this study, we used spatial analysis, geostatistics, and Bayesian linear regression to investigate the invasion dynamics and colonization processes of this pest in New Jersey. We present the results of monitoring *H. halys* from 51 to 71 black light traps that were placed on farms throughout New Jersey from 2004 to 2011 and examined relationships between total yearly densities of *H. halys* and square hectares of 48 landscape/land use variables derived from urban, wetland, forest, and agriculture metadata, as well as distances to nearest highways. From these analyses we propose the following hypotheses: (1) *H. halys* density is strongly associated with urban developments and railroads during its initial establishment and dispersal from 2004 to 2006; (2) *H. halys* overwintering in multiple habitats and feeding on a variety of plants may have reduced the Allee effect, thus facilitating movement into the southernmost regions of the state by railroads from 2005 to 2008; (3) density of *H. halys* contracted in 2009 possibly from invading wetlands or sampling artifact; (4) subsequent invasion of *H. halys* from the northwest to the south in 2010 may conform to a stratified-dispersal model marked by rapid long-distance movement, from railroads and wetland rights-of-way; and (5) high densities of *H. halys* may be associated with agriculture in southern New Jersey in 2011. These landscape features associated with the invasion of *H. halys* in New Jersey may predict its potential rate of invasion across the USA and worldwide.

## Introduction

The brown marmorated stink bug, *Halyomorpha halys* (Stål) (Hemiptera: Pentatomidae), is native to China, Japan, and Korea and is an invasive agricultural pest in the mid-Atlantic United States of America (USA), that was introduced around 1996 into Allentown, Pennsylvania (USA) [17]. Since then it has been found in over 41 states, causing significant reductions in agricultural yields and has become a homeowner nuisance [25]. It is highly polyphagous and able to feed on a wide variety of both agricultural and non-agricultural plants. Some of these plants susceptible to injury include field crops, tree and small fruit, vegetables, and wild and ornamental plants. *H. halys* became a major pest of multiple crops, and its potential for damage is increasing as its range in the USA broadens. Due to high populations in 2010, damage from *H. halys* resulted in over $37 million of loss to mid-Atlantic apples and vegetables [2], [24]. Monitoring of this pest and other Pentatomoidea pest species has been conducted using baited pyramid traps, visual sampling, and black lights [25], [27], [37], [38]. Current control relies on frequent insecticide applications, impacting established integrated pest management programs.

*H. halys* overwinters in forested areas and structures (D–H. Lee, personal comm.). In forested areas, dead trees and standing trees provide overwintering habitat. In urban areas, human-made structures such as residential and commercial buildings also provide overwintering habitat. In some cases, large numbers of overwintering *H. halys* can be found [19]. The ability to overwinter in forests and structures coupled with its wide host range have allowed *H. halys* to spread and establish populations throughout New Jersey and beyond to other states.

Because *H. halys* can overwinter in a variety of habitats, are polyphagous, and have a high rate of dispersion [25], [26], [35], [36], their invasion dynamics may be difficult to examine using conventional statistical models. Thus, spatial analysis (i.e. Geographic Information Systems or GIS) and geostatistics may be more appropriate in examining these dynamics. These analyses have allowed researchers to correlate spatially referenced data with landscape factors through space and time. In fact, the spatial context of individuals and populations has become an important aspect of understanding ecological processes involving insects [28]. This ability is especially useful when characterizing susceptible habitats and analyzing survey data. For example, Shepherd et al. [41] were able to determine how forest type and climate correlated with outbreaks of *Orgyia pseudotsugata* (McDunnough) (Douglas-fir tussock moth) by using historical maps of defoliation, forest type, and climate maps. Similarly, GuoJun et al. [15] investigated the invasion dynamics and dispersal processes of *Lissorhoptrus oryzophilus* Kuschel (rice water weevil) in China using historical maps of pest invasion and distribution of infested rice.

The diverse New Jersey landscape allows for a unique opportunity to investigate invasion dynamics of *H. halys* and landscape features that are facilitating this invasion using spatial analytical tools. New Jersey is comprised of a range of landscapes, from highly urbanized areas, farmland, coastal beaches and marshes to grasslands and wetland forests [33]. Over the past two decades, urban development has rapidly changed New Jersey’s landscape and caused extensive fragmentation [16]. This changing mosaic has implications on the spread of a highly polyphagous species like *H. halys.* For example, knowledge of the distribution of *H. halys* and the understanding of its invasion dynamics and colonization processes influenced by landscape parameters would help predict areas susceptible to *H. halys* infestation. In addition, growers in affected areas could target control efforts in areas likely to have elevated populations due to landscape composition and how ecosystem processes such as disturbances caused by pest species are affected by landscape. Examination of stink bug population spatial dynamics has been facilitated by the use of GIS and the compilation of spatial data. These studies have been beneficial in revealing surrounding habitats near Hawaiian macadamia nut orchards that supported high densities of the southern green stink bug *Nezara viridula* (L.) [20]; edge effects of *N. viridula* in peanut-cotton landscapes [47]; population dynamics of *N. viridula* in corn, cotton, and peanut landscapes [46]; and patchy distributions for several pest species of Pentatomidae, such as *N. viridula*, *Euschistus servus* (Say), *Oebalus pugnax* (F.), and *Thyanta custator* (F.) in South Carolina wheat fields [40]. However, few studies on *H. halys*’ landscape ecology exist. Kiritani [22] showed that increases in *H. halys* populations in Japan seem to have risen after the planting of conifer plantations following World War II. Zhu et al. [53] developed a model predicting the potential distribution of *H. halys* with respect to climatic factors, but was not able to predict distribution based on landscape features.

Although, some studies [22], [53] have examined environmental factors that may facilitate the dispersal of *H. halys* over geographical areas, a state-wide landscape analysis of *H. halys* is lacking. Here, we analyzed our vast dataset of the invasion dynamics of *H. halys* in New Jersey to develop a robust model (i.e. hypothesis) that elucidates the movement of this insect over a large geographical area that is based on natural (e.g., forest, wetland, and grassland habitats) and anthropogenic (e.g., houses, silos, and roads) factors. Nielsen and colleagues [38] identified the yearly population increase of *H. halys* to be 75% based on black light trap catches. The incorporation of landscape features and biological information into a predictive model could be readily tested in other states in the USA and other infested countries. Lastly, these data could offer novel control strategies of this pest insect, for example landscapes that are more susceptible to infestation.

Already, the state of New Jersey has the longest historical data set in the USA of *H. halys* collected from black light traps distributed throughout the state. Although specimens were collected from black light traps beginning in 1999 through 2011, only specimens captured from 2004 to 2011 were used in our analysis because active monitoring of this pest was conducted during these years. In using this dataset, the focus of our study was to 1) examine the invasion dynamics, establishment, and colonization processes of *H. halys* throughout New Jersey from 2004 to 2011 using geostatistical analysis in ArcGIS; 2) determine which environmental factors are facilitating this invasion using Bayesian linear regression analysis; 3) from these results develop a series of hypotheses of *H. halys* dispersal; and 4) discuss landscapes that are susceptible by this pest insect.

## Materials and Methods

**Figure 1:**
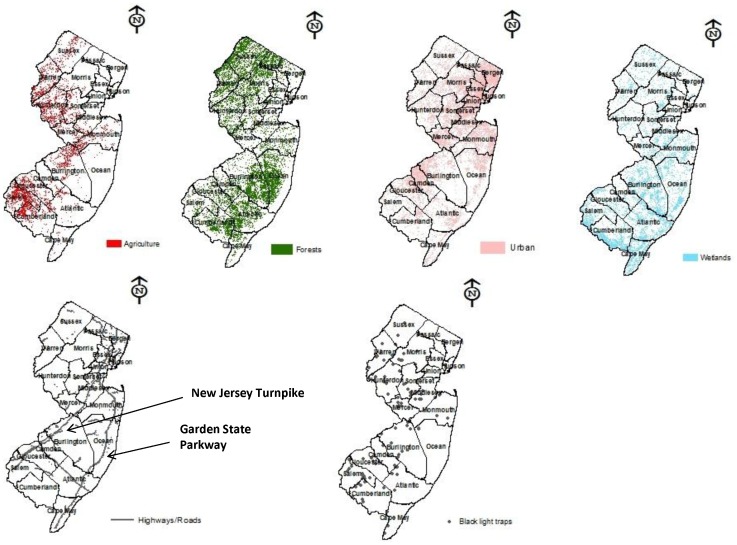
Maps of New Jersey displaying the five main landscape/land use features used in the analysis, including agriculture, forests, urban/residential, wetlands, and major highways and roads; the New Jersey Turnpike and The Garden State Parkway are shown in grey; and distribution of black light traps throughout New Jersey are also provided.

Insect monitoring with back light traps (110V Gempler’s, Madison WI) occurred on 51–71 farms throughout New Jersey and each trap was geo-referenced using a handheld Trimble GeoExplorer II (Trimble Navigation Ltd. Sunnyvale, Calif) GPS unit with 2 to 5m (6.6 to 16.4ft) accuracy after differential correction [18]. Black light traps were placed in open area of the farms in front of sheds or silos. All samples were done on private land as part of the Rutgers Vegetable IPM program. Rutgers has the permission to take these samples. Traps were selected because *H. halys* is attracted to traps in large numbers [38] and provides a standardized sampling effort. Traps were distributed throughout the agricultural areas of the state with fewer traps located in urbanized areas in the northeast, southeastern regions of New Jersey characterized by the sandy soils of the Pinelands (i.e. heavily forested area of coastal plain occupying seven counties in southern New Jersey), and the Atlantic coast (see Figure 1 placement of traps). Monitoring of these insects initiated in 2004 and continues to the present. Because data are still being recorded and analyzed, we focused on *H. halys* collected from 2004 to 2011, an important period of its invasion throughout the state [38].

Insects were collected bi-weekly from May to October through the Rutgers Cooperative Extension Vegetable IPM program for each of these years [35]. Adult stink bug specimens were sorted from these traps and identified to species according to [17], [32]. Less than 1% of specimens did not have their gender determined. Voucher specimens are currently housed at the Rutgers Entomology Insect Museum in New Brunswick, New Jersey [38]. Weekly counts of male and female *H. halys* for each year and farm were merged because we were interested in the effects of land use/land cover factors on the total population of *H. halys* throughout New Jersey. These data were compiled into a matrix using Microsoft Excel and imported into ArcGIS 10 [9] and linked to the corresponding trap locations.

### Environmental Variables

We selected environmental features (i.e. landscape/land use) that might potentially affect population dynamics of *H. halys* in New Jersey [5], [48], [53]. These features are classified into five types of landscape/land cover features, which include agriculture, urbanization, wetlands, forests, and distance to nearby roads and highways. Landscape/land use features within the Pinelands and along the Atlantic coast were excluded in the analysis because no traps were placed in these habitats. These five main types of features were obtained by acquiring GIS digital files from the New Jersey Department of Environmental Protection (NJDEP) GIS Land use/Land cover dataset from 2007 [34], [9] (see Figure 1). These files were selected because they represent the most current datasets produced by NJDEP. These data were taken from aerial photography captured in the spring of 2007.

We merged datasets from every county within New Jersey to create individual maps representing the five main types of landscape/land use features using the merger function in ArcMap 10. Trap locations were plotted on these maps; buffers of 2 km were placed around each of the traps, representing the total hectares *H. halys* may inhabit [52]; and square acreage of 48 land use/land cover variables within the main landscape/land use features (see Table S1 for list of variables and acreage) was calculated using the calculate geometry function in ArcMap 10. Lastly distance, in meters, from each trap to the nearest highways was calculated in ArcMap 10. Square hectares of all 48 variables, distances to nearest highway, and total population of *H. halys* for each trap were entered into Excel for statistical analysis.

## Data Analysis

### Kernel Density Estimation

Kernel Density Estimation (KDE) was implemented to examine the overall invasion dynamics and colonization of *H. halys* using the geographical information systems software ArcMap 10. KDE uses probability statistics (i.e. kernel function) to calculate the estimated density of a population around the actual population data points producing a fitted, smooth, tapered surface that connects all data points used in the analysis [9], [42]. We selected KDE because this analysis performs well with small amounts of data, they are robust to autocorrelation, and results are in a utilization distribution (i.e. UD; A UD is a grid where the value for each cell represents the probability of the taxa occurring in that cell) rather than a simple distribution range outline [21].

KDEs were derived from *H. halys* density collected from 2004 through 2011 and bandwidths (i.e. smoothing parameter). In calculating these bandwidths, we randomly measured distances between 20 traps, and then averaged those distances. A bandwidth specifies the maximum distance at which data points (i.e. traps) are distributed over the study area or the search radius of the Kernel Density function [9].

### Hot-Spot Analysis

Hot-Spot Analysis was used to detect statistically significant populations of *H. halys* over New Jersey from 2004 to 2011. This was done to provide information on the overall dispersal and distribution of *H. halys* throughout New Jersey and provide insight on the relative rate of spread of *H. halys* populations across the New Jersey landscape. This analysis was used to compliment KDE because it can detect statistically significant (i.e., Z-scores) high (i.e., hot spot) and/or low (cold spot) clustering of *H. halys* populations. To be a statistically significant hot spot, a farm with a high density of *H. halys* has to be surrounded by neighboring farms with high densities of *H. halys*. The local sum of *H. halys* in a farm and its neighbors is then compared to the sum of all farms for each year; when the local sum is very different from the expected local sum, and that difference is too large to be the result of random chance, a statistically significant Z-score will result [9]. Z-scores are standard deviations, associated with p-values found in the tails of the normal distribution.

### Bayesian Analysis

Bayesian linear regression was implemented to examine which of the 48 landscape/land use factors are facilitating the invasion, establishment, and dispersal of *H. halys* populations in New Jersey from 2004 to 2011 using the statistical software WinBUGS (Bayesian inference Using Gibbs Sampling; [43]). Bayesian linear regression was selected over univariate [e.g. analysis of variance (ANOVA)] and multivariate [e.g. principal component analysis (PCA)] statistical methods because it is a powerful and robust methodology that allows the observer to combine total *H. halys* population data with additional, independently available information (e.g. landscape/land use variables; the prior) to produce a full probability distribution (posterior distribution) of the relationships between *H. halys* populations and various landscape/land use factors; allows one to make inferences based on small sample sizes; and it appropriately models uncertainty on those relationships [6], [8], [10], [11], [23]. Moreover, the Bayesian methodology allows us to ask directly how probable is the hypothesis (e.g. Ho: acreage of different agriculture and urban landscape/land use factors will influence *H. halys* populations over time and space), given the data; whereas, conventional statistical methods lack this advantage. The following Bayesian regression model (Equation 1) was fitted to the hectare and total *H. halys* data, using reversible jump MCMC methods:

**Equation 1.** Bayesian Regression Model used in the analysis.
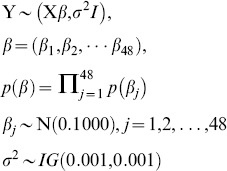



Reversible jump MCMC (RJMCMC) was selected over other Markov chain algorithms because this algorithm allows for sampling of posterior distributions that are generated from an unequal numbers of traps (i.e., samples) [13], [14], [29]. RJMCMC computations are generated by computing and converging on the most optimal posterior distributions. These computations are accomplished by fitting complex models (e.g. linear regression model) to our *H. halys* population and landscape/land use data. For relationships between *H. halys* and landscape/land use data to be considered informative, the posterior distribution of that relationship must be 0.05 or greater [i.e., a high posterior probability (i.e., α-level = 0.05), which indicates a statistically significant relationship between total *H. halys* population and a particular environmental variable]. RJMCMC sampled from 1,000,000 iterations and variable selection, which reflected a strong association (i.e., high probability) with the *H. halys* data, was based on the final 500,000 iterations [29]–[31].

The notations of *σ* ^2^ and *β* in the model above (Equation 1) represent the prior distributions on our parameters, where *IG* (inverse-gamma distribution) represents the marginal posterior distribution for the unknown variance of a normal distribution when an uninformative prior is used. These priors were chosen to be non-informative or vague because we are primarily interested in understanding what relationships are informative between landscape/land use variables and total *H. halys* populations [23]. Other notations in the model include the dependent variables (i.e., Y_1_, Y_2_,…,Y_8_). More specifically, Y_n_ represents the total *H. halys* populations collected from each farm from 2004 through 2011. Lastly, X represents the independent variables or covariates (i.e., 48 landscape/land use variables).

## Results

**Figure 2:**
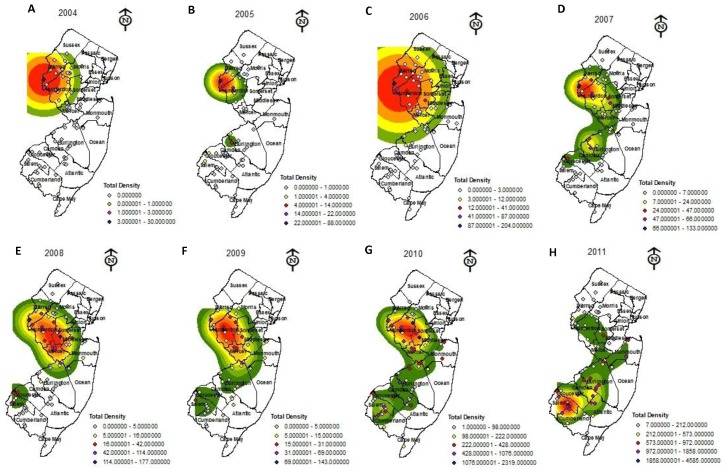
Kernel Density Estimation (KDE) graphs of the density of *Halymorpha halys* captured from black light traps placed throughout New Jersey from (A) 2004, (B) 2005, (C) 2006, (D) 2007, (E) 2008, (F) 2009, (G) 2010, (H) 2011. KDE are based on actual and predicted density of *H. halys* where green reflects lowest population density, orange moderate to high population density, and red predicts highest population of *H. halys*. Total density of *H. halys’* for year black lights were monitored is also provided.

**Figure 3:**
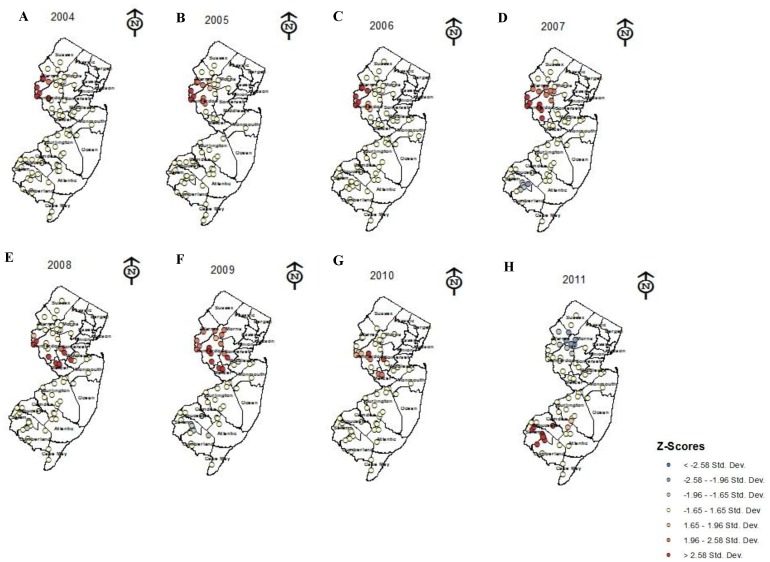
Hot-spot/cold-spot analysis graphs of the densities of *Halyomorpha halys* captured from black light traps placed throughout New Jersey from (A) 2004, (B) 2005, (C) 2006, (D) 2007, (E) 2008, (F) 2009, (G) 2010, (H) 2011. Z-scores are standard deviations, associated with p-values found in tails of the normal distribution, with red and orange reflecting high density of *H. halys* that are significantly clustering with one another and light to dark blue reflecting low densities of *H. halys* that are significantly clustering with one another.

The KDE analysis, generated from data collected in 2004, displayed high densities of *H. halys* in Warren County (northwest region), New Jersey, with no populations occurring in the remainder of the state (Figure 2A). A statistically significant hot-spot was also detected in this county (Figure 3A). In 2005, populations of *H. halys* almost tripled in size to 192 individuals (season total *H. halys* numbers across all black light traps; Table S1). In addition to this increase in population size, the KDE analysis displayed two populations (Figure 2B). The first and largest of these populations was still found in the northwest portion of the state but had subsequently dispersed east and south into most of Warren and Hunterdon Counties. The second population was detected further south in Burlington and Camden Counties. However, the hot-spot analysis only detected one statistically significant hot-spot in Warren and Hunterdon Counties (Figure 3B).

In 2006, the KDE analysis continued to display high densities of *H. halys* in the northwest region of New Jersey (Table S1, Figure 2C). Moreover, probability estimates from the KDE analysis (Figure 2C) showed that these insects spread further east into Middlesex County and south into Mercer, Somerset, and Morris Counties, although populations detected in Burlington and Camden Counties from 2005 were absent. A similar trend of high density and evidence of range expansion of these insects was detected in the hot-spot analysis, but only in Warren and Hunterdon Counties (Figure 3C).

By 2007, *H. halys* populations increased from a total of 473 individuals observed in 2006 to a total of 766 individuals found in 2007, despite a slight decrease in six black light traps between these years (Table S1). Probability estimates from the KDE analysis (Figure 2D) showed that this insect continued to disperse further east into Middlesex and Monmouth Counties, as well as further south into Burlington, Camden, Gloucester, and Salem Counties; and thus yielding a 50% increase in range expansion for *H. halys* from 2006 to 2007 (see Figures 2C–D). This range expansion observed in 2007 is reflected in the hot-spot analysis, which detected an expansion of high clusters of *H. halys* in Warren, Hunterdon, and Morris Counties, as well as detecting statistically low populations of *H. halys* (i.e., contraction of populations) in Salem and Cumberland Counties (Figure 3D).

In 2008 we observed an increase in population size from 766 in 2007 to a total of 1283 in 2008 (Table S1), but a retraction in the distribution of *H. halys* from Camden County (Figure 2E) observed in 2007 (Figure 2D). Although we observed a contraction in range distribution of this insect in 2008, despite an increase in the number of black light traps used, the overall distribution in 2008 was similar, in that *H. halys* density was concentrated in the northwest and spreading south into the center of New Jersey; a small population was present in southern portion of the state (Salem and Gloucester Counties); and the hot-spot analysis detected two high densities of *H. halys* (Figure 3E). One of these hot-spots was still in the northwest and a second hot-spot was observed in the southeast. This result corroborates with the KDE analysis, in that high densities of this insect are spreading further south into the state. Lastly, a cold spot was detected in Salem and Cumberland Counties, suggesting a contraction in *H. halys* density in southern New Jersey (Figure 3E). This trend is similar to the cold spot observed in 2007 (Figure 3D).

By 2009, the KDE analysis (Figure 2F) displayed densities of *H. halys* dispersing into 12 counties in New Jersey from 2008 (Figure 2E); extending their range east into Ocean County; further south in Burlington County; and further extending their range of an isolated population established in 2007 (Figure 3D) in the southernmost part of New Jersey (Salem, Cumberland, and Gloucester Counties; Figure 2F). Also, these data (Figure 2F) indicate that *H. halys* continue to spread further south New Jersey. This trend is reflected in the hot-spot analysis of 2009 (Figure 3F) which displays an increase in hot-spots in the eastern, southern, and northern parts of New Jersey compared to 2008 (Figure 3E). Although, *H. halys* expanded its distribution range the total density of this insect decreased to 677 from 1283 in 2008 (Table S1).

In 2010, we observed a rapid increase in the distribution range and density of *H. halys* from 12 counties in 2009 (Figure 2F) to 14 counties in 2010 (Figure 2G, Figure 3). Other trends observed include a high density of *H. halys* still being maintained in northwest New Jersey but continuing to move south, particularly in Hunterdon and Somerset Counties (Figure 2G) from Warren County observed in previous years (Figures 2A–F); a continued increase in density of *H. halys* in the southernmost portion of New Jersey, particularly in Salem and Cumberland Counties; and additional populations of these insects observed east into Monmouth and Ocean Counties, central in Burlington County, and south into Camden, Gloucester, and Atlantic Counties, as well as in southeast portion of New Jersey (i.e., Cape May County; Figure 2G). This continued dispersal into the southern region of the state is exhibited in the hot-spot analysis (Figure 3G) showed a decreased in the number of hot-spots observed in 2009 (Figure 3F) with no high density of *H. halys* occurring in Warren County as in previous years (Figure 3A–F) and an absence of cold-spots in the southern part of New Jersey (Figure 3G).

Lastly in 2011, we observed an inverse of the trend encountered in 2004 (Figure 2A) in which high densities of *H. halys* occurred in the southernmost part of state, such as Salem, Cumberland, and Gloucester Counties whereas only a few populations of this insect were found in Warren, Hunterdon, and Somerset Counties (Figure 2H). This trend conforms to the hot-spot analysis, which detected significant clusters of *H. halys* in the southernmost part of New Jersey and cold-spots (i.e., contraction) in the northwest part of New Jersey (Figure 3H). Lastly, while the overall distribution range of this insect slightly decreased from 12 counties in 2010 to 11 counties in 2011, the density had doubled (Figure 2H).

### Environmental Factors

**Figure 4:**
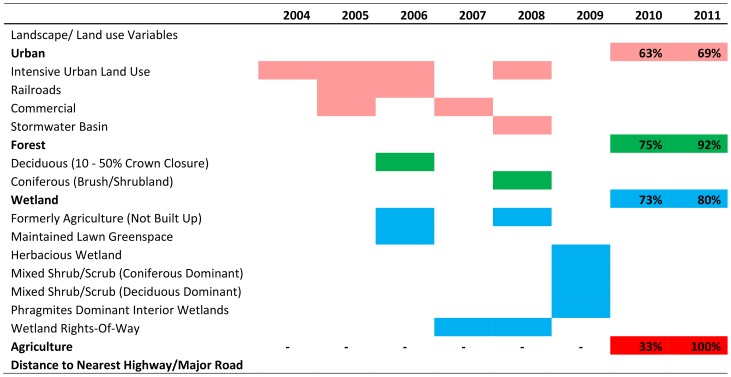
Summary table of landscape/land use variables that displayed significant positive relationship with densities of *Halyomorpha halys* using Bayesian linear regression analysis. Pink represents urban landscape/land use variables; green represents forest landscape/land use variables; blue represents wetland landscape/land use variables; and red represents agricultural landscape/land use variables. In 2010 and 2011 we calculated the total number of these relationships observed.

The Bayesian linear regression identified 41 out of the total 48 landscape/land use variables that displayed a statistically significant relationship with densities of *H. halys* from 2004 through 2011 (Table 1). More specifically, in 2004 we observed intensive land use, for example, communications and utility buildings, suburban communities, and parking lots, displayed a positive relationship with densities of *H. halys*. As the populations dispersed east and south in 2005 (Figure 2B) we observed strong associations with not only intensive land use, but also with commercial land use (e.g. strip malls) and railroads (Table 1, Figure 4). As *H. halys* populations in 2006 increased in density and continued to disperse east and south (Figure 2C), we found this insect displayed strong associations with urban factors, such as railroads, and other landscape/land use features that include deciduous forests and wetlands (e.g. fallow agricultural land and wetland communities either natural or planted that are maintained in residential, commercial, and or industrial areas; Figure 4).

**Table 1:** Probability distributions calculated from total yearly densities of *Halyomorpha halys* captured from black light traps placed on farms from 2004 to 2011 using Bayesian linear regression and the reversible jump MCMC algorithm.

Landscape/Land use Variables	2004	2005	2006	2007	2008	2009	2010	2011
CROPLAND AND PASTURELAND	0.00016	0.00029	0.0003	0.0017	0.0015	0.0001	0.0123	**0.9439**
ORCHARDS/VINEYARDS/NURSERIES/HORTICULTURAL AREAS	0.00008	0.00023	0.0006	0.0005	0.0005	0.0003	0.0058	**0.0946**
OTHER AGRICULTURE	0.00030	0.00144	0.0023	0.0036	0.0063	0.0017	**0.9635**	**0.1713**
Distance	0.00000	0.00003	0.0000	0.0000	0.0000	0.0000	0.0003	0.0041
CONIFEROUS BRUSH/SHRUBLAND	0.00079	0.00226	0.0052	0.0049	**0.1062**	0.0065	**0.9998**	**0.1312**
CONIFEROUS FOREST (>50% CROWN CLOSURE)	0.00041	0.00092	0.0033	0.0024	0.0031	0.0018	0.0289	**0.0816**
CONIFEROUS FOREST (10–50% CROWN CLOSURE)	0.00173	0.00672	0.0148	0.0108	0.0146	0.0074	**0.1421**	**0.4825**
DECIDUOUS BRUSH/SHRUBLAND	0.00186	0.01068	0.0195	0.0060	0.0052	0.0046	**0.0544**	**0.1655**
DECIDUOUS FOREST (>50% CROWN CLOSURE)	0.00008	0.00015	0.0003	0.0003	0.0009	0.0002	0.0100	0.0223
DECIDUOUS FOREST (10–50% CROWN CLOSURE)	0.00138	0.01235	**0.0946**	0.0142	0.0051	0.0101	**0.0808**	**0.0927**
MIXED DECIDUOUS/CONIFEROUS BRUSH/SHRUBLAND	0.00038	0.00085	0.0027	0.0051	0.0382	0.0063	**0.3388**	**0.0905**
MIXED FOREST (>50% CONIFEROUS WITH >50% CROWN CLOSURE)	0.00031	0.00122	0.0031	0.0030	0.0053	0.0024	0.0157	**0.0495**
MIXED FOREST (>50% CONIFEROUS WITH 10–50% CROWN CLOSURE)	0.00150	0.00530	0.0147	0.0112	0.0232	0.0112	**0.1337**	0.3840
MIXED FOREST (>50% DECIDUOUS WITH >50% CROWN CLOSURE)	0.00030	0.00132	0.0030	0.0036	0.0081	0.0056	0.0166	**0.0657**
MIXED FOREST (>50% DECIDUOUS WITH 10–50% CROWN CLOSURE)	0.00122	0.00533	0.0133	0.0111	0.0133	0.0154	**0.0854**	**0.3872**
OLD FIELD (<25% BRUSH COVERED)	0.00051	0.00155	0.0043	0.0034	0.0050	0.0026	**0.0517**	**0.1280**
PLANTATION	0.00142	0.00409	0.0207	0.0137	0.0120	0.0053	**0.1484**	**0.2501**
RAILROADS	0.00576	**0.06867**	**0.2849**	0.0495	0.0339	0.0074	**0.1610**	**0.2605**
ATHLETIC FIELDS (SCHOOLS)	0.00099	0.00357	0.0068	0.0131	0.0138	0.0058	**0.1077**	**0.3121**
CEMETERY	0.00194	0.00504	0.0171	0.0127	0.0188	0.0045	**0.1558**	**0.2217**
COMMERCIAL/SERVICES	0.00679	**0.06826**	0.0144	**0.7959**	0.0025	0.0048	**0.0708**	**0.0539**
INDUSTRIAL	0.00040	0.00152	0.0029	0.0024	0.0077	0.0009	0.0280	**0.0509**
MAJOR ROADWAY	0.00086	0.00418	0.0045	0.0457	0.0219	0.0025	**0.1000**	**0.1890**
OTHER URBAN OR BUILT-UP LAND	**0.46699**	**0.37560**	**0.1806**	0.0307	**0.0548**	0.0429	**0.3694**	0.0498
RECREATIONAL LAND	0.00219	0.00858	0.0113	0.0040	0.0387	0.0031	0.0274	0.0453
RESIDENTIAL, HIGH DENSITY OR MULTIPLE DWELLING	0.00069	0.00143	0.0036	0.0020	0.0035	0.0011	0.0358	**0.0585**
RESIDENTIAL, RURAL, SINGLE UNIT	0.00016	0.00026	0.0006	0.0008	0.0041	0.0021	0.0156	0.0224
RESIDENTIAL, SINGLE UNIT, LOW DENSITY	0.00015	0.00072	0.0011	0.0016	0.0011	0.0006	0.0203	0.0327
RESIDENTIAL, SINGLE UNIT, MEDIUM DENSITY	0.00011	0.00055	0.0007	0.0024	0.0006	0.0003	0.0270	0.0175
STORMWATER BASIN	0.00263	0.00662	0.0258	0.0148	**0.5166**	0.0067	**0.5515**	**0.2693**
TRANSPORTATION/COMMUNICATION/UTILITIES	0.00415	0.00383	0.0097	0.0135	0.0078	0.0056	**0.0830**	**0.2349**
UPLAND RIGHTS-OF-WAY DEVELOPED	0.00381	0.01138	0.0394	0.0234	**0.7640**	0.0389	**0.5384**	**0.4097**
UPLAND RIGHTS-OF-WAY UNDEVELOPED	0.00168	0.00583	0.0242	0.0106	0.0078	0.0036	**0.2672**	**0.3562**
AGRICULTURAL WETLANDS (MODIFIED)	0.00010	0.00045	0.0010	0.0011	0.0011	0.0011	0.0221	**0.0768**
CONIFEROUS WOODED WETLANDS	0.00047	0.00170	0.0043	0.0044	0.0078	0.0265	**0.0510**	**0.1391**
DECIDUOUS SCRUB/SHRUB WETLANDS	0.00137	0.00396	0.0171	0.0083	0.0102	0.0072	**0.0905**	**0.2712**
DECIDUOUS WOODED WETLANDS	0.00063	0.00317	0.0039	0.0100	0.0072	0.0004	**0.5888**	0.0238
DISTURBED WETLANDS (MODIFIED)	0.00064	0.00250	0.0059	0.0089	0.0069	0.0077	**0.0902**	**0.1200**
FORMER AGRICULTURAL WETLAND (SOME SHRUBS, NOT BUILT-UP)	0.00785	0.02948	**0.0574**	0.0409	**0.0518**	0.0227	**0.4830**	**0.4956**
HERBACEOUS WETLANDS	0.00113	0.00339	0.0079	0.0060	0.0079	**0.2885**	**0.0889**	**0.2401**
MANAGED WETLAND IN BUILT-UP MAINTAINED REC AREA	0.00282	0.01193	0.0265	0.0238	0.0246	0.0143	**0.2547**	**0.3276**
MANAGED WETLAND IN MAINTAINED LAWN GREENSPACE	0.02417	0.03335	**0.0536**	0.0285	0.0457	0.0231	**0.3317**	**0.5552**
MIXED SCRUB/SHRUB WETLANDS (CONIFEROUS DOM.)	0.00282	0.00974	0.0185	0.0167	0.0431	**0.5924**	**0.2557**	**0.3274**
MIXED SCRUB/SHRUB WETLANDS (DECIDUOUS DOM.)	0.00083	0.00299	0.0061	0.0046	0.0077	**0.0509**	**0.1157**	**0.1749**
MIXED WOODED WETLANDS (CONIFEROUS DOM.)	0.00019	0.00077	0.0016	0.0019	0.0025	0.0085	0.0188	0.0470
MIXED WOODED WETLANDS (DECIDUOUS DOM.)	0.00031	0.00108	0.0026	0.0045	0.0061	0.0337	0.0291	**0.0646**
PHRAGMITES DOMINATE INTERIOR WETLANDS	0.00096	0.00276	0.0067	0.0088	0.0095	**0.0520**	**0.0779**	**0.1623**
WETLAND RIGHTS-OF-WAY	0.00352	0.00963	0.0208	**0.0502**	**0.0836**	0.0253	**0.4247**	**0.6943**

For relationships between *H. halys* and landscape/land use data to be considered informative, the posterior distribution of that relationship must be 0.05 or greater [(i.e. α-level = 0.05), indicating a statistically significant relationship].

In 2007 as *H. halys* populations continued to move further south they displayed strong positive relationships with commercial structures and wetlands rights-of-way (former wetlands converted to road with some shrub vegetation; Figure 4). By 2008 and 2009, this insect dispersed further south (Figures 2E–F) and showed positive relationships with coniferous forests, and wetland landscapes (e.g., wetland right-of-way, *Phragmites*, fallow agriculture land, and mixed wetlands), and less so with urban factors, for example intensive urban land use and storm water basins (i.e., surface water collection site, associated with new commercial and residential areas), compared to 2005 and 2006 (Table 1, Figure 4). Lastly, from 2010 and 2011, *H. halys* displayed positive significant relationships with the majority (63–92%) of New Jersey landscape features examined. Among these landscape/land use features, agricultural variables, for example vineyards and orchards (Table 1, Figure 4) displayed positive relationships with this insect. Moreover, from 2010 to 2011 we observed a 70% increase in relationships between agriculture variables and *H. halys*. Although, we observed a trend in associations between *H. halys* and various landscape/land use features, relationships between this insect pest and distance to nearest highways and major roads from 2004 through 2011 were absent (Figure 4).

## Discussion

Understanding landscape factors (i.e., both natural and anthropogenic) that facilitate the spread and establishment of invasive pests is a critical element in invasion biology [50], [39]. The invasion into New Jersey by *H. halys* and the spread of this pest throughout the state has created an excellent and unique opportunity to determine factors affecting the invasion dynamics and colonization process of this insect pest, as well as provide insight into developing novel strategies to control this pest. The invasion of *H. halys* can be divided into four phases: 1) initial establishment and dispersal, 2) range expansion, 3) potential lag phase or contraction in population growth, and 4) exponential growth.

### First Phase: Initial Establishment and Dispersal

Within the first two years, from 2004 to 2005, the initial dispersal of *H. halys* began in the northwestern portion of New Jersey (Warren and Hunterdon Counties; Figures 2A). These populations were in close proximity to where this insect pest was first detected in Allentown, PA to the east (Somerset and Mercer Counties). A small population in the south (Burlington and Camden Counties) was later detected in 2005 (Figure 2B). Accompanying this initial dispersal we observed an increase in density from 2004 to 2005 (Table S1). Landscape/land use factors displaying positive relationships with this dispersion include: intensive urban land use, commercial developments (e.g., residential houses, strip malls, and supermarkets), and railroads (Figure 4). These factors may have been instrumental during the initial dispersion and establishment because these commercial and residential developments have provided adequate protection and increased the overwintering survival of *H. halys* [22], [19].

Another possible explanation for the patterns observed from 2004 to 2005 is the Allee effect. The Allee effect is a positive relationship between the number of individuals in a population and their fitness [1]. Meaning that an individual of a species that is subject to an Allee effect will suffer a decrease in some aspect of its fitness when population density is low [1], [44], [45], [3]. The population of *H. halys* is hypothesized to be severely, genetically bottlenecked and arising from a single introduction with a small propagule size [51] as small as two females. Theoretically, this would cause a significant reduction in fitness caused by inbreeding depression through Allee effect. However, as evidenced by our results, *H. halys* appears to have successfully overcome the Allee effect and spread at a rate of 2.84 farms per year with a 75% annual population increase [38]. The correlation between urban and residential developments during this establishment phase may have reduced the Allee effect of *H. halys*, thus facilitating a higher rate of establishment and dispersion. A similar pattern was observed in French Polynesia for the glassy-winged sharpshooter, *Homalodisca vitrepinnis* (Germar). For example, islands experiencing the early stages of invasion by *H. vitrepinnis* displayed high densities in urbanized areas with substantially lower abundances on remote islands further away from the main population [39].

The small satellite population of *H. halys* observed in Burlington and Camden Counties may also be a result of the Allee effect (Figure 2B) as has been shown in other insects [39]. Brown et al. [4] observed that many species of passerine birds displaying a reduced Allee effect have relatively clustered distribution with many sites of low abundance and few hot-spots of high local abundance.

Human-transportation activity (i.e., the movement of plants and contaminated cargo via ships, cars, boats, and rails) may also explain the increase in *H. halys* density and its establishment and range expansion in northwestern New Jersey. These activities can deliberately move material while simultaneously and unintentionally moving pest species, such as *H. halys* [12], [39]. Of these activities, we observed a positive relationship between *H. halys* density and railroads in 2004 and 2005 (Figure 4). This suggests that railroads, also present in northwestern New Jersey (Warren and Hunterdon Counties), may have eased barriers to establishment resulting in the movement of this insect into new habitats. The association with railroads has been demonstrated in the establishment and dispersion of other insect pests, such as the glassy-winged sharpshooter in French Polynesia [39] and the rice water weevil in China [15]; although additional monitoring and analysis is required to adequately elucidate the relationship between *H. halys* and railroads.

In addition, these railroads and urban/residential developments may be surrounded by high host-plant diversity for *H. halys*. Two such hosts are *Paulownia tomentosa* (Thunb.) Sieb. and Zucc. ex Steud. (i.e., princess tree) and *Ailanthus altissima* (Mill.) Swengel (i.e., tree of heaven), natives to China [7], [35], [25]. These tree species are ornamentals, found in parks, gardens, and can be found in highly disturbed environments (e.g. intensive urban development and potentially near railroads). Because of the potential close proximity of these trees to railroads and urban/residential developments the following effect may have been produced: *P. tomentosa* and *A. altissima* are acting as a sink for *H. halys*, which could accelerate their invasion into urban/residential developments thereby increasing its abundance and reducing the Allee effect; and *H. halys* could readily move from *P. tomentosa* and *A. altissima* to nearby rail cars.

Lastly, orchard trees may be facilitating the movement of this pest insect to rail cars. These orchard trees are another potential source of establishment and dispersion because they are known hosts of *H. halys* [26]. Moreover, these orchards occupy approximately 434 hectares in Warren and Hunterdon Counties [49], which were the first counties to harbor this pest insect. Despite this evidence, additional investigations are needed to examine the associations between orchards, railroads, and *H. halys* in early establishment.

### Second Phase: Population Growth and Range Expansion

After the initial establishment and dispersal of *H. halys* in 2004 and 2005, we observed rapid population growth and range expansion from 2006 to 2008 (Figure 2D–F). This growth and range expansion was associated with an increase in the insect’s relationship with urban, forest, and wetland landscape/land use variables than the previous years (Table 1, Figure 4). The agriculture/urban interface, which is common in New Jersey, may have facilitated population growth of *H. halys* by offering agricultural and cultivated host plants for development and natural and human-made overwintering structures. The association with human habitat may be specifically important and has been suggested to increase overwintering survival relative to pentatomid species that overwinter in natural habitats [22]. As a consequence, we hypothesize that populations of *H. halys* may have rapidly increased, overcoming a critical minimum density threshold, followed by the establishment of new populations observed in eastern and southern New Jersey (Figures 2C–E, 3C–E), and thus reducing a possible Allee effect.

In addition to overcoming the Allee effect, the long distance movement from northwestern New Jersey to the southernmost portion of the state from 2006 to 2008 (Figures 2C–E) may be a consequence of stratified diffusion. Stratified diffusion refers to the movement of an invasive species by rapid long-distance transport and local diffusion around a point of infestation [39]. Long-distance movement may have been facilitated by rail cars to central New Jersey in 2006 (Figure 4). Moreover, these railroads are located in areas where high densities of *H. halys* have been observed in 2006 (Figure 2C). In 2007 and 2008 long distance-movement from central New Jersey to the southernmost part of the state may be attributed to close proximity of commercial developments (e.g., strip malls, motels, and gas stations) acting as stepping-stones for invasion and fruit tree orchards or other adjacent suitable habitats near wetland rights-of-way (Figure 4). It should be noted that no relationships were detected between *H. halys* and highways/roads because black lights may have been too far away from this pathway.

The second component of stratified dispersion is local diffusion which occurs when a nascent colony becomes established and begins to expand naturally. This diffusion of *H. halys* after the long distance movement, appearing in 2007 (3 years after initial establishment to the southernmost region of New Jersey), may have been facilitated by encountering new habitats for overwintering, such as deciduous forests [i.e., dead, standing trees (D–H. Lee, unpublished data)]. Other factors facilitating this diffusion may include suitable host plants near fallow agriculture that has undergone succession into wetlands, and the increased hectares of orchard trees located in the southernmost region of New Jersey, such as Gloucester and Burlington Counties [49].

### Phase 3: Potential Spurious Lag Phase

Following this population growth and expansion phase observed from 2006 to 2008 (Figures 2C–E), was a subsequent reduction in population size of *H. halys* in 2009 (Figure 2F, Table S1). This reduction coincides with a decrease in the number of positive significant relationships observed between *H. halys* and landscape/land use variables (Table 1, Figure 4). More specifically, only wetland variables, such as herbaceous wetlands, mixed shrub/scrub deciduous and coniferous wetlands, and Phragmites wetlands (Table 1, Figure 4) displayed significant positive relationships with this insect. These results suggest these wetland habitats could be barriers for mate finding of *H. halys* [45], although additional data are needed to examine the impact of these wetlands on the reproduction of this insect. Another more probable explanation in the decrease of *H. halys* observed in 2009 may be a sampling artifact, caused by 51 black light traps used in 2009 compared to 71 black light traps used in 2008. This potential spurious result is further supported in Nielsen et. al [38] study, which showed a continuing increase in populations of *H. halys* from 2004–2011 in New Jersey when black light traps are kept constant.

### Phase 4: Exponential Growth

An exponential growth phase was observed from 2010 to 2011 (Table S1), as well as *H. halys* possibly dispersing and/or populations increasing from the north to the south (Figures 1G–H, 2G–H). This pattern was associated with all the main landscape/land use categories, with the exception of major highways and roads (Table 1, Figure 4). Positive density increases of *H. halys* and their subsequent expansion into the southernmost part of the state may be attributed to higher acreage of suitable habitats, in particular orchards. This was supported by a positive relationship observed between orchards and *H. halys* (Table 1). The high acreage of orchard trees in southern New Jersey [49], which are important hosts of this insect [25], [26], [36], may have supported high densities of *H. halys*. Although, continued monitoring in and near these orchards is needed to increase confidence in this trend.

Although there are many positive aspects of our dataset, our conclusions are dependent upon the location of the black light traps themselves. In this study, the black light traps are always placed in a vegetable farm and thus we have minimal data in non-agricultural areas such as densely populated urban areas in northeastern New Jersey, the Pinelands, and coastal regions. Additional black light traps in these habitats are needed to provide a more complete picture of the invasion and colonization dynamics of *H. halys* in New Jersey and other regions with diverse landscape/land use features.

## Conclusions

We identified four phases of *H. halys* invasion in New Jersey from this study: (1) initial establishment (2004) and dispersal (1 year after invasion was being monitored in New Jersey) of the invasive population is confined to urban/residential developments and railroads, populations densities are low (92–189 individuals), and there is absence of this pest on the rest of the state, with the exception of a satellite population in the south. (2) An invasion of intermediate duration (2–3 years after becoming established in northwestern New Jersey) is characterized by an expansion of its range further east and south, and exhibiting relationships with urban, forest, and wetland habitats. Pest density in these habitats increased from 473 to 1283. (3) A potential contraction in population growth in 2009 after approximately 5 years from the initial establishment of this pest, although this pattern may be attributed to reduction in black light traps. Associations with wetland habitats in the northwestern region of New Jersey were present. (4) Finally, after 8 years of monitoring the invasive *H. halys* population reached high densities of 17690 in 2010 and 34241 in 2011 with high abundance in urban, wetland, forest, and agricultural habitats. Human-mediated long-distance dispersal events (e.g., railroads, wetland rights-of-way, and potentially trucks) may have facilitated the movement of this pest. In addition, high adaptability to novel overwintering sites and alternative host plants, as well as areas with large hectares of agriculture in the northwest and particularly in the south of New Jersey may have contributed to the high density, establishment, and dispersal of this pest. Stricter regulations preventing plant movement combined with monitoring and implementation of integrated pest management strategies near railroads, wetland rights-of-way, urban/residential developments, and orchards with known high density of *H. halys* may be needed to greatly reduce the spread of this pest throughout New Jersey and other states with documented invasions of *H. halys*.

## Supporting Information

Table S1 **Density of** ***H. halys*** **captured from black light traps placed on farms throughout New Jersey from 2004 to 2011.** Latitude and longitude are provided for each of these traps. Total densities are recorded for each year and NA (not available) reflects the traps not used for that particular year
(DOCX)
