# Supplementary material for: Landscape Factors Facilitating the Invasive Dynamics and Distribution of the Brown Marmorated Stink Bug, Halyomorpha halys (Hemiptera: Pentatomidae), after Arrival in the United States
Source: PLoS One. 2014 May 1;9(5):e95691. doi: 10.1371/journal.pone.0095691 (PMC4006787; doi:10.1371/journal.pone.0095691)
Supplement: Table S1 — Density of H. halys captured from black light traps placed on farms throughout New Jersey from 2004 to 2011. Latitude and longitude are provided for each of these traps. Total densities are recorded for each year and NA (not available) reflects the traps not used for that particular year (DOCX) [file pone.0095691.s001.docx]

**Table S1.** Density of *H. halys* captured from black light traps placed on farms throughout New Jersey from 2004 to 2011. Latitude and longitude are provided for each of these traps. Total densities are recorded for each year and NA (not available) reflects the trap not used for that particular year.

| **Farms** | **Latitude** | **Longitude** | **BMSB 2004** | **BMSB 2005** | **BMSB 2006** | **BMSB 2007** | **BMSB 2008** | **BMSB 2009** | **BMSB 2010** | **BMSB 2011** |
| --- | --- | --- | --- | --- | --- | --- | --- | --- | --- | --- |
| Farm A | -74.80331 | 40.93844 | 0 | 0 | 0 | 0 | 2 | NA | 1 | 50 |
| Farm B | -74.61383 | 40.15209 | 0 | NA | 0 | 12 | 12 | 4 | 249 | 282 |
| Farm C | -75.3719 | 39.36221 | 0 | 0 | NA | NA | NA | NA | NA | NA |
| Farm D | -75.37192 | 39.36221 | 0 | 2 | NA | 27 | 37 | 7 | NA | 523 |
| Farm E | -74.68059 | 41.16576 | NA | NA | 0 | 0 | 0 | NA | NA | 7 |
| Farm F | -75.05748 | 40.80948 | 0 | 0 | 0 | 2 | 9 | NA | 196 | NA |
| Farm G | -75.04823 | 40.81966 | 1 | 3 | 0 | NA | 0 | 4 | 8 | 95 |
| Farm H | -74.98141 | 40.95202 | 0 | 0 | 1 | 6 | 1 | NA | NA | 21 |
| Farm I | -75.23202 | 39.57814 | NA | NA | 0 | 2 | 0 | NA | NA | NA |
| Farm J | -74.79661 | 40.07235 | 0 | 1 | 0 | 5 | 5 | 10 | 265 | 468 |
| Farm K | -75.04823 | 40.81966 | NA | NA | 10 | 2 | 3 | 2 | 256 | NA |
| Farm L | -75.41156 | 39.65434 | 0 | 1 | 0 | 3 | 12 | 21 | 511 | 141 |
| Farm M | -74.83835 | 40.73244 | 0 | 0 | 0 | NA | 0 | 1 | NA | 364 |
| Farm N | -75.23609 | 39.34068 | 0 | 0 | 0 | 0 | 1 | 10 | 138 | 1858 |
| Farm O | -75.20955 | 39.54821 | 0 | 0 | 0 | NA | NA | NA | NA | NA |
| Farm P | -75.09211 | 39.73531 | 0 | 0 | 0 | 10 | 14 | NA | NA | 108 |
| Farm Q | -74.70701 | 40.77613 | 0 | 0 | NA | NA | 0 | 8 | 1 | 89 |
| Farm S | -74.72956 | 40.77990 | 0 | 4 | 12 | 63 | 1 | 7 | 62 | 140 |
| Farm T | -74.99232 | 40.01331 | NA | 22 | 28 | 60 | 73 | 20 | 498 | 330 |
| Farm U | -74.94092 | 40.60949 | 0 | 0 | 0 | NA | NA | NA | NA | NA |
| Farm V | -75.29295 | 39.49914 | 0 | 0 | NA | NA | NA | NA | NA | NA |
| Farm W | -74.62950 | 40.13709 | 0 | 0 | 0 | 13 | 72 | 23 | 305 | 663 |
| Farm X | -74.92277 | 40.53692 | 0 | 2 | 6 | 6 | 15 | 6 | 113 | NA |
| Farm Y | -74.47469 | 40.37614 | NA | NA | NA | 0 | 1 | NA | NA | NA |
| Farm Z | -74.53069 | 40.37816 | 0 | 0 | 0 | 3 | 5 | 1 | 135 | 92 |
| Farm AA | -74.50404 | 40.86787 | 0 | 0 | 0 | 1 | 9 | NA | 1 | NA |
| Farm AB | -75.05818 | 39.70368 | 0 | 0 | 0 | 1 | 1 | 0 | 408 | 1515 |
| Farm AC | -74.94865 | 39.45564 | 0 | 1 | 0 | NA | 1 | 2 | NA | 144 |
| Farm AD | -74.90032 | 39.21004 | 0 | 0 | 0 | NA | 0 | NA | 85 | 54 |
| Farm AE | -74.81237 | 39.68682 | 0 | 0 | 0 | 1 | 0 | 2 | 221 | 1452 |
| Farm AF | -75.13662 | 39.55761 | 0 | 1 | 0 | 2 | NA | NA | NA | NA |
| Farm AG | -74.23802 | 40.20758 | 0 | 0 | 0 | 2 | 16 | 8 | 259 | 1224 |
| Farm AH | -74.93775 | 38.98944 | 0 | 0 | NA | NA | NA | NA | NA | NA |
| Farm AI | -74.66976 | 40.53377 | NA | NA | NA | 43 | 104 | NA | NA | NA |
| Farm AJ | -74.69191 | 40.84455 | 0 | 0 | 1 | 0 | 5 | 6 | 4 | 77 |
| Farm AK | -74.84462 | 39.60601 | NA | 0 | 0 | 0 | 0 | NA | 34 | 812 |
| Farm AL | -74.64517 | 40.09881 | 0 | 0 | NA | NA | NA | NA | NA | NA |
| Farm AN | -74.89752 | 39.06274 | 0 | NA | 0 | 3 | 8 | 13 | 136 | 328 |
| Farm AM | -74.59801 | 40.44311 | 0 | 0 | 0 | 0 | 0 | 1 | 175 | 134 |
| Farm AO | -74.84990 | 40.82815 | 0 | 0 | 2 | 24 | 20 | 2 | NA | 212 |
| Farm AP | -74.79651 | 39.67524 | 0 | 0 | NA | NA | 1 | 8 | 62 | NA |
| Farm AQ | -74.77613 | 39.59872 | 0 | 0 | 0 | 2 | 0 | 2 | 363 | 1422 |
| Farm AR | -74.76926 | 39.67058 | 0 | 0 | NA | 2 | NA | NA | NA | NA |
| Farm AS | -74.63420 | 40.48170 | NA | NA | 22 | 26 | 138 | 21 | 2319 | 689 |
| Farm AT | -74.75319 | 40.30891 | 0 | 0 | 2 | 15 | 114 | NA | NA | NA |
| Farm AU | -74.76116 | 40.39650 | 0 | NA | NA | NA | NA | NA | NA | NA |
| Farm AV | -74.76539 | 39.77606 | 0 | NA | 0 | 2 | 2 | NA | 93 | 765 |
| Farm AW | -74.75114 | 39.80413 | 0 | 0 | NA | 6 | 0 | 9 | 98 | 1293 |
| Farm AX | -74.45471 | 40.37842 | NA | NA | 0 | NA | NA | NA | NA | NA |
| Farm AY | -75.22312 | 39.30995 | 0 | 0 | 0 | 0 | 0 | 1 | 127 | NA |
| Farm AZ | -74.60248 | 40.41069 | NA | NA | NA | NA | NA | NA | NA | NA |
| Farm BA | -74.71786 | 40.30520 | 0 | 1 | 0 | 12 | 24 | 69 | 291 | 203 |
| Farm BB | -75.10958 | 40.63007 | 0 | 14 | 87 | 18 | 33 | 5 | 343 | 120 |
| Farm BC | -75.07314 | 40.60727 | NA | NA | NA | NA | NA | NA | 172 | 177 |
| Farm BD | -74.75948 | 40.78732 | 0 | 0 | 0 | 2 | 1 | NA | 2 | 134 |
| Farm BE | -75.44083 | 39.62501 | 0 | 1 | 0 | 2 | 20 | NA | NA | NA |
| Farm BF | -74.21177 | 40.41855 | 0 | NA | 0 | 2 | 0 | 7 | 245 | 719 |
| Farm BG | -74.80202 | 39.43700 | NA | NA | NA | NA | NA | NA | 66 | 311 |
| Farm BH | -74.84889 | 39.92163 | 0 | 0 | 3 | 3 | 1 | 5 | 152 | 803 |
| Farm BI | -75.15763 | 40.58499 | 3 | 13 | 41 | 41 | 26 | 4 | 209 | 130 |
| Farm BJ | -74.45668 | 40.42477 | 0 | NA | 2 | 3 | NA | 8 | 175 | 82 |
| Farm BK | -74.52960 | 40.73383 | 0 | 0 | 2 | 1 | 4 | NA | NA | 119 |
| Farm BL | -75.23321 | 39.76058 | 0 | 1 | NA | NA | NA | NA | NA | NA |
| Farm BM | -74.42783 | 40.46226 | NA | NA | NA | NA | 24 | NA | NA | NA |
| Farm BN | -74.49131 | 40.07330 | 0 | NA | 1 | 2 | 10 | 15 | 180 | 320 |
| Farm BO | -74.48918 | 40.06747 | 0 | NA | 0 | 0 | 0 | 3 | 222 | 442 |
| Farm BP | -74.76746 | 41.03576 | 0 | 0 | 0 | 1 | 2 | NA | NA | 26 |
| Farm BQ | -74.26110 | 40.39546 | NA | NA | 0 | 0 | 3 | 7 | 132 | 75 |
| Farm BR | -74.74884 | 40.66512 | 0 | 2 | 1 | 11 | 24 | 28 | 1629 | 206 |
| Farm BS | -75.41856 | 39.74823 | 0 | 1 | 1 | 3 | 20 | 2 | 279 | 864 |
| Farm BT | -74.77023 | 40.34111 | 0 | 1 | 1 | 20 | 42 | 26 | 252 | 330 |
| Farm BU | -75.15901 | 40.70102 | 30 | 88 | 204 | 133 | 177 | 48 | 1076 | 432 |
| Farm BV | -74.96020 | 40.55756 | 0 | NA | NA | NA | NA | NA | NA | NA |
| Farm BW | -75.24056 | 39.60217 | NA | NA | NA | NA | NA | 2 | 11 | 461 |
| Farm BX | -74.95809 | 40.77447 | 0 | 4 | 6 | 35 | 19 | NA | NA | 148 |
| Farm BY | -74.72468 | 40.33193 | 0 | 0 | 1 | 4 | 70 | 31 | 273 | 136 |
| Farm BZ | -75.20760 | 39.51950 | NA | NA | 0 | 0 | 2 | 3 | 237 | 1756 |
| Farm CA | -75.25545 | 39.48128 | 0 | 1 | 0 | 3 | 1 | 2 | 75 | NA |
| Farm CB | -74.89565 | 40.43010 | 0 | 0 | 0 | 7 | 20 | 7 | 675 | 414 |
| Farm CC | -75.12204 | 39.76894 | NA | 1 | 0 | NA | NA | NA | NA | NA |
| Farm CD | -75.32213 | 39.46325 | NA | NA | NA | 0 | 0 | NA | 428 | 1164 |
| Farm CE | -75.23202 | 39.57814 | 0 | 0 | 0 | NA | 0 | 5 | 656 | 4585 |
| Farm CF | -75.22106 | 39.57748 | NA | 0 | 2 | NA | NA | NA | NA | NA |
| Farm CG | -74.96100 | 40.56120 | 0 | 9 | 23 | 47 | 57 | 143 | 1817 | 573 |
| Farm CH | -74.69270 | 40.53220 | 0 | NA | NA | NA | 1 | 14 | 357 | 550 |
| Farm CI | -74.96934 | 39.89154 | 0 | 13 | 12 | 66 | 9 | 9 | 536 | 625 |
| Farm CJ | -74.60112 | 40.06908 | 0 | 0 | 0 | NA | NA | NA | NA | NA |
| Farm CK | -74.70663 | 39.83522 | 0 | 0 | 0 | 4 | 1 | 2 | 7 | 669 |
| Farm CL | -75.23458 | 39.55200 | NA | NA | NA | NA | 0 | NA | NA | 972 |
| Farm CM | -74.09092 | 40.17681 | NA | NA | 0 | NA | NA | NA | NA | NA |
| Farm CN | -75.41156 | 39.65434 | 0 | 2 | 2 | 2 | 2 | 28 | 1 | 13 |
| Farm CO | -75.38828 | 39.68049 | NA | NA | NA | NA | 8 | NA | 69 | 1330 |
| **Total Density** |  |  | **92** | **189** | **473** | **766** | **1283** | **672** | **17690** | **34241** |
